# Novel Murine Infection Models Provide Deep Insights into the “Ménage à Trois” of *Campylobacter jejuni*, Microbiota and Host Innate Immunity

**DOI:** 10.1371/journal.pone.0020953

**Published:** 2011-06-15

**Authors:** Stefan Bereswill, André Fischer, Rita Plickert, Lea-Maxie Haag, Bettina Otto, Anja A. Kühl, Javid I. Dashti, Andreas E. Zautner, Melba Muñoz, Christoph Loddenkemper, Uwe Groß, Ulf B. Göbel, Markus M. Heimesaat

**Affiliations:** 1 Department of Microbiology and Hygiene, Charité - University Medicine Berlin, Berlin, Germany; 2 Department of Pathology/Research Center ImmunoSciences (RCIS), Charité - University Medicine Berlin, Berlin, Germany; 3 Department of Medical Microbiology, University Medical Center Göttingen, Göttingen, Germany; Albany Medical College, United States of America

## Abstract

**Background:**

Although *Campylobacter jejuni*-infections have a high prevalence worldwide and represent a significant socioeconomic burden, it is still not well understood how *C. jejuni* causes intestinal inflammation. Detailed investigation of *C. jejuni*-mediated intestinal immunopathology is hampered by the lack of appropriate vertebrate models. In particular, mice display colonization resistance against this pathogen.

**Methodology/Principal Findings:**

To overcome these limitations we developed a novel *C. jejuni*-infection model using gnotobiotic mice in which the intestinal flora was eradicated by antibiotic treatment. These animals could then be permanently associated with a complete human (hfa) or murine (mfa) microbiota. After peroral infection *C. jejuni* colonized the gastrointestinal tract of gnotobiotic and hfa mice for six weeks, whereas mfa mice cleared the pathogen within two days. Strikingly, stable *C. jejuni* colonization was accompanied by a pro-inflammatory immune response indicated by increased numbers of T- and B-lymphocytes, regulatory T-cells, neutrophils and apoptotic cells, as well as increased concentrations of TNF-α, IL-6, and MCP-1 in the colon mucosa of hfa mice. Analysis of MyD88^−/−^, TRIF^−/−^, TLR4^−/−^, and TLR9^−/−^ mice revealed that TLR4- and TLR9-signaling was essential for immunopathology following *C. jejuni*-infection. Interestingly, *C. jejuni*-mutant strains deficient in formic acid metabolism and perception induced less intestinal immunopathology compared to the parental strain infection. In summary, the murine gut flora is essential for colonization resistance against *C. jejuni* and can be overcome by reconstitution of gnotobiotic mice with human flora. Detection of *C. jejuni*-LPS and -CpG-DNA by host TLR4 and TLR9, respectively, plays a key role in immunopathology. Finally, the host immune response is tightly coupled to bacterial formic acid metabolism and invasion fitness.

**Conclusion/Significance:**

We conclude that gnotobiotic and “humanized” mice represent excellent novel *C. jejuni*-infection and -inflammation models and provide deep insights into the immunological and molecular interplays between *C. jejuni*, microbiota and innate immunity in human campylobacteriosis.

## Introduction

In industrialized countries *Campylobacter jejuni* is among the most frequent causative agents of bacterial enteritis [1]. The Gram-negative pathogen is transmitted via the food chain from farm animals to humans by the ingestion of undercooked meat, non-pasteurized milk, and water [2], [3]. After infection *C. jejuni* colonizes the human distal small intestine and colon. Clinical symptoms include abdominal pain, fever, myalgia, and watery or bloody diarrhea. After active invasion of colonic epithelial cells, *C. jejuni* induces mucosal inflammation characterized by neutrophil infiltration, crypt abscesses, focal ulcerations, and plasma cell proliferation [4]–[6]. Although the vast majority of infections are self-limited, in some cases infection might result in chronic sequelae such as Reiter's syndrome, reactive polyarthropathy, and Guillain-Barré syndrome [2], [7].

Whereas colonization factors of *C. jejuni* are well known [8], the molecular mechanisms underlying immunopathology of *C. jejuni*-infection in the host are still poorly understood. Insights into *C. jejuni* - host interactions are limited due to the scarcity of suitable experimental *in vivo* models [9]. While chickens are well suited to investigate colonization, vertebrate models using newborn pigs, weanling ferrets, gnotobiotic canine pups, and primates have numerous limitations including high costs, handling issues, and a lack of reproducibility [9], [10]. Mice are highly convenient for the study of bacterial pathogenicity and can help to overcome several of these limitations. Murine models of *C. jejuni*-infection, however, have the disadvantage of sporadic colonization and/ or absence of clinical disease manifestations [1]. This is in part due to the colonization resistance against *C. jejuni* displayed by conventional mice with a normal commensal microflora. The use of germfree animals or mice with a limited gut flora has alleviated these shortcomings with varying degrees of success [10], [11]. It was demonstrated earlier that *C. jejuni* colonized the entire gastrointestinal (GI) tract of isolator-raised germfree mice and induced clinical signs of disease including granulocyte infiltrates, bloody diarrhea, and humoral immune responses, reproducibly occurring after infection [12]–[16]. However, germfree mice have an abnormal development of gut-associated lymphoid tissue [17], [18]. Therefore, germfree mice might not represent a suitable experimental model of *C. jejuni*-infections in humans due to the lack of an intact innate immune system. Furthermore, mice deficient in the central innate immunity adaptor protein MyD88 essential in Toll-like receptor (TLR) signaling were stably colonized by *C. jejuni* but did not develop intestinal inflammation [19]. Interaction of *C. jejuni* with individual TLRs, namely TLR2, TLR4, and TLR9 recognizing bacterial lipoproteins (LP), lipopolysaccharide (LPS), or CpG-DNA, respectively, still await detailed investigation particularly *in vivo* and in the intestines *in situ*. In many *in vitro* studies, however, TLR signaling could be demonstrated to be involved in *C. jejuni*-induced immune responses. Whereas *C. jejuni* bacteria were not recognized by human TLR5 [20], live and lysed *C. jejuni* cells activated TLR2, TLR4 and TLR9 via MyD88 in different human, murine or avian cell lines [21]–[25]. The role of TLR9 signaling in human *C. jejuni* enteritis is not clear yet. *C. jejuni* activated avian, but not human TLR9 transiently expressed in a transfected cancer cell line *in vitro* [21]. However, the role of TLR9 in *C. jejuni* enteritis has not been investigated *in vivo* so far. For proper LPS-mediated TLR4 activation, the adapter proteins MyD88 or TRIF are essentially required [26]. In recent studies, IL-10 deficient mice harboring a conventional gut flora developed significant signs of intestinal immunopathology following *C. jejuni*-infection [27]–[29]. In addition, enteritis and bacterial translocation were also observed in TLR4^−/−^ IL-10^−/−^ double deficient mice [29]. Taken together, these results point towards pivotal functions of the host-specific gut microflora and the innate immune system in both, infection control and inflammation.

In order to optimize murine models for *C. jejuni* enteritis mimicking human immunopathology, we used our gnotobiotic murine model in which the intestinal flora was completely eradicated by quintuple antibiotic treatment [30]–[32]. Then, we reconstituted these germfree mice with a complete human gut flora to display the human intestinal environment in mice with a fully developed immune system. The results obtained with *C. jejuni*-infected gnotobiotic or “humanized” mice presented here prove for the first time that the host specific gut flora plays a crucial role in colonization resistance against *C. jejuni*. Furthermore, we demonstrate that TLR4- and, strikingly, TLR9-mediated signaling are essentially involved in *C. jejuni*-induced immunopathology. Lastly, we show that the host responses to *C. jejuni* infection are tighly coupled to the pathogen's formic acid metabolism and perception essential for cell invasion fitness by analyzing the respective deletion mutants in our novel *in vivo* models. Thus, our results presented here indicate for the first time that gnotobiotic mice reconstituted with human gut flora provide deep insights into the “ménage à trois” of *C. jejuni*, human commensal gut flora and the host innate immune system.

## Results

### Gnotobiotic mice as a novel model for studying *C. jejuni* - host interactions

Given that the commensal murine gut microbiota is essential for the resistance against *C. jejuni* colonization and thus suitable mouse models of *C. jejuni*-infection are scarce, we established a novel murine experimental system to investigate the interplay (“ménage à trois”) of the gut microbiota and innate immune system on the host side with the pathogen after oral *C. jejuni*-infection and permanent intestinal colonization. For this purpose, we generated gnotobiotic mice by quintuple antibiotic treatment for six weeks (refer to [30]). It was essential to handle these animals under strictly sterile conditions throughout the entire antibiotic treatment and experimental procedures in order to prevent from secondary contaminations. Results from cultural and molecular analyses confirmed that these gnotobiotic mice were completely free of any culturable and non-culturable bacteria (**Fig. S1**). Similar to isolator-raised germfree animals, the antibiotics-treated mice developed a mega-cecum that is a characteristic morphologic indicator for a sterile gut.

We determined the colonization capacity of *C. jejuni* in gnotobiotic mice after antibiotic treatment as compared to mice harboring a conventional gut flora following peroral infection with the *C. jejuni* reference strains ATCC 43431 [33], [34], 81–176 [35], [36] and B2 [37]. These strains originate from humans with severe enteritis, are invasive *in vitro* and were extensively investigated in earlier studies. *C. jejuni* 81–176 carries a virulence plasmid that encodes components of a type IV protein secretion machinery [38].

The results confirmed that conventional mice bred in our SPF facilities display a strong colonization resistance, as at the time of necropsy at day (d) 12 post infection (p.i.) *C. jejuni* irrespective of the strain used could not be detected in fecal samples in the majority of the animals (**Fig. S2**). Kinetic studies revealed that *C. jejuni* was expelled from the GI tract already within 48 hours after infection (data not shown). Strikingly, colonization resistance was completely abrogated in gnotobiotic mice generated by quintuple antibiotic treatment as all three *C. jejuni* strains colonized at high concentrations of 10^9^–10^10^ colony forming units (CFU) per gram fecal sample at day 12 p.i. (**Fig. S2**).

**Figure 1:**
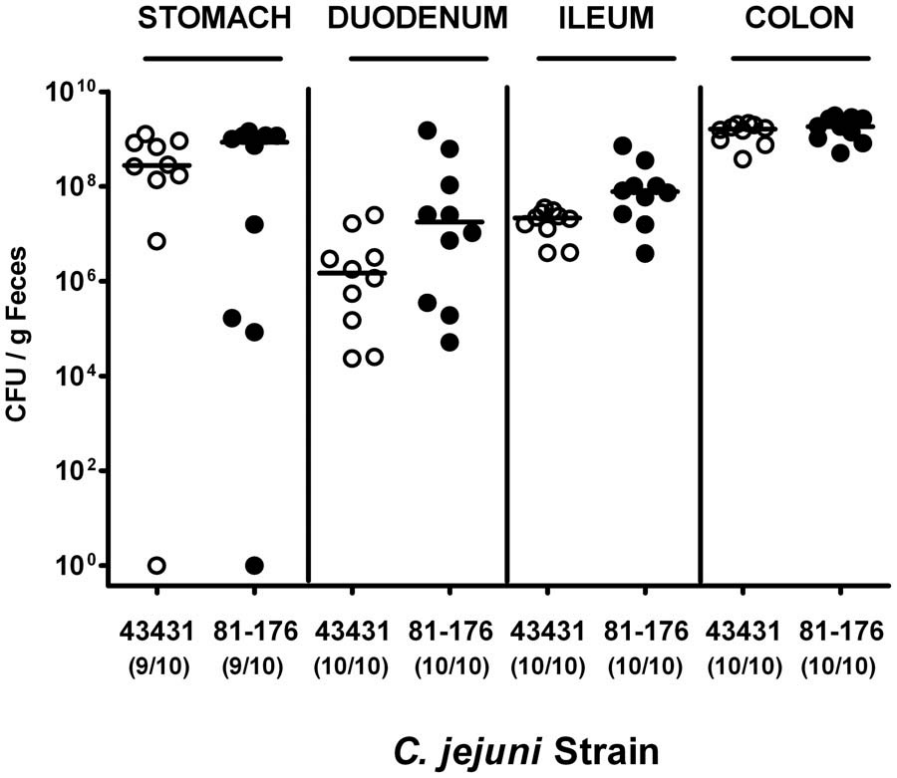
*C. jejuni* colonization along the gastrointestinal tract of gnotobiotic mice. Gnotobiotic mice generated by antibiotic gut decontamination were orally infected with *C. jejuni* strains ATCC 43431 (open circles) or 81–176 (closed circles) as described in methods. The pathogen densities in distinct compartments of the gastrointestinal tract were determined by quantification of live *C. jejuni* in luminal samples taken from stomach, duodenum, ileum, and colon at day 12 p.i. by cultural analysis (CFU, colony forming units). Numbers of animals harboring *C. jejuni* out of the total number of analyzed animals are given in parentheses. Medians (black bars) are indicated. Data shown were pooled from three independent experiments.

In addition, *C. jejuni* densities within the GI tract of gnotobiotic mice were analyzed in more detail (**Fig. 1**). Following peroral infection, *C. jejuni* ATCC 43431 and 81–176 readily colonized the stomach, duodenum, ileum and colon at comparable levels with the highest bacterial loads in the large intestine reaching >10^9^ CFU per gram luminal content at d12 p.i.. Kinetic experiments revealed that in gnotobiotic animals, *C. jejuni* colonization was stable throughout a time period of more than 100 days p.i. (data not shown), indicating that this model is well suited to examine long-term evolution and host-adaptation of the pathogen *in vivo*.

### TLR4 and TLR9 play an important role in *C. jejuni* immunopathology

In order to investigate the role of TLR4 and TLR9 signaling in the development of immunopathology after stable *C. jejuni* colonization *in vivo*, we generated gnotobiotic mice deficient in TLR4, TLR9, MyD88 or TRIF by antibiotic treatment.

**Figure 2:**
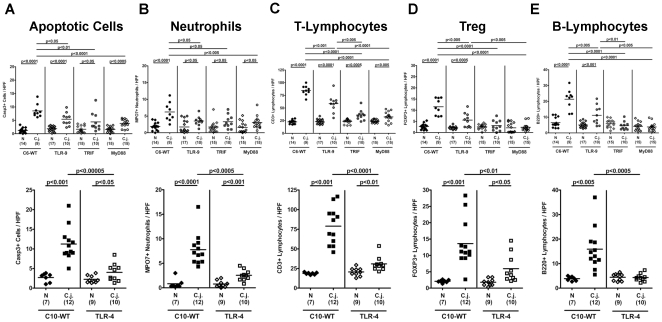
Impact of host bacterial sensing on immunopathology in the colon following *C. jejuni*-infection. Gnotobiotic wildtype (WT; C57BL/10 (C10) or C57BL/6 (C6) as indicated), TLR4^−/−^ (lower panel), TLR9^−/−^, TRIF^−/−^, and MyD88^−/−^ (upper panel) mice generated by antibiotic gut decontamination were orally infected with *C. jejuni* strain ATCC 43431. The average numbers of apoptotic cells (positive for caspase-3, panel **A**), neutrophilic granulocytes (neutrophils, positive for MPO-7, panel **B**), T-lymphocytes (positive for CD3, panel **C**), regulatory T-cells (Tregs, positive for FOXP3, panel **D**) and B-lymphocytes (positive for B220, panel **E**) from at least six high power fields (HPF, 400× magnification) per animal were determined microscopically in immunohistochemically stained colon sections. Numbers of animals of the respective genotype analyzed are given in parentheses. Means (black bars) and levels of significance (*P*-values) as compared to the respective infected WT control group (determined by the Student's *t*-test) are indicated. Data shown were pooled from three independent experiments.

At day 12 following peroral infection with *C. jejuni* ATCC 43431, significant histopathological changes in colon sections of wildtype (WT) mice such as loss of goblet cells, crypt elongation, and immune cell infiltration could be observed which were less pronounced in infected TLR4-deficient mice (Figure S3). In order to more specifically and precisely analyze distinct immune cell responses in the inflamed colon *in situ* and to quantitate the degree of histopathology we performed immunohistochemical stainings of colon section. Given that apoptosis is commonly used as a diagnostic marker in the histopathological evaluation and grading of intestinal diseases, we quantitatively determined apoptotic cells and, in addition, the influx of neutrophils acting as inflammatory effector cells within the colon mucosa following *C. jejuni*-infection. Twelve days p.i., *C. jejuni* ATCC 43431 colonization densities were comparable within the respective segments of the GI tract (stomach, duodenum, ileum, and colon) irrespective of the genotype of mice under investigation with highest bacterial counts in the colon (approximately 10^9^ CFU/ g luminal colon content; data not shown). Gnotobiotic mice deficient in TLR4, TLR9, TRIF or MyD88, however, displayed significantly less inflammatory responses compared to respective wildtype controls as indicated by lower numbers of Caspase3^+^ apoptotic and MPO7^+^ neutrophilic granulocytes in the colon *in situ* (**Fig. 2 A, B**). Furthermore, we observed significantly lower numbers of CD3^+^ T-lymphocytes, FOXP3^+^ regulatory T-cells (Tregs), and B220^+^ B-lymphocytes within the colonic mucosa of TLR4^−/−^, TLR9^−/−^, TRIF^−/−^, and MyD88^−/−^ infected mice compared to WT mice at d12 p.i. (**Fig. 2 C–E**). Interestingly, Treg and B-lymphocyte counts of infected TRIF^−/−^ and MyD88^−/−^ animals did not differ when compared to non-infected controls (Fig. 2 D, E). In addition, B220^+^ cells within the colon mucosa of TLR4^−/−^ mice did not increase within 12 days following *C. jejuni*-infection (**Fig. 2 E**). These results hightlight the gnotobiotic mouse model following antibiotic treatment as a well-suited vertebrate model for the study of *C. jejuni* colonization and pathogen-host interaction mounting in immunopathological responses similar to those seen in humans with *Campylobacter* enteritis. Strikingly, we demonstrate for the first time *in vivo* that MyD88- and TRIF-dependent sensing of *Campylobacter*-LPS and -CpG-DNA via TLR4- and TLR9-signaling is essentially involved in *C. jejuni*-mediated immunopathology.

### Generation of mice reconstituted with a complete human gut flora

**Figure 3:**
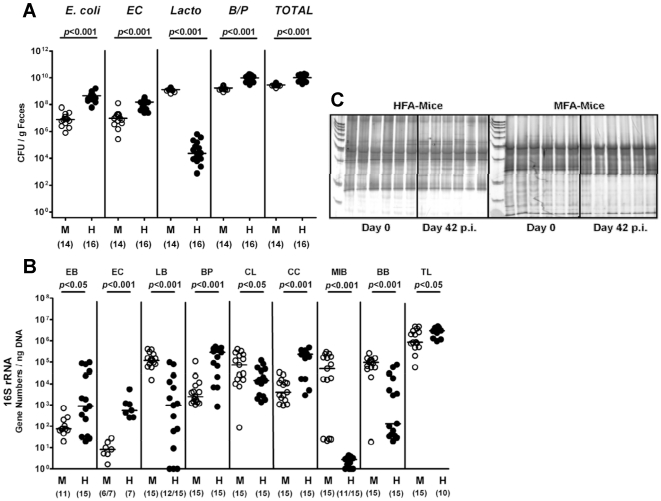
Microflora analysis of gnotobiotic mice with human or murine gut flora. Gnotobiotic mice generated by antibiotic gut decontamination were recolonized with a human (HFA, H) or murine (MFA, M) microflora as described (see methods). Main gut bacterial groups were quantified by culture and molecular analysis of fecal samples before and after *C. jejuni*-infection (d42 p.i.). **(A) Culture.** Total bacterial counts (*TOTAL*) and numbers of *E. coli*, enterococci (*EC*), Lactobacilli (*Lacto*) and *Bacteroides/ Prevotella* spp. (*B/P*) were determined in feces samples of MFA (M) and HFA (H) mice right before *C. jejuni*-infection (day 0) by detection of colony forming units (CFU) per gram feces on appropriate culture media (see methods). Bacterial species were identified by biochemical analysis and reconfirmed by comparative sequence analyses of 16S rRNA genes. Numbers of animals harboring the respective bacterial species are given in parentheses. Medians and significance levels (*P*-values) determined by Mann-Whitney-U test are indicated. Data shown were pooled from three independent experiments. **(B) RT-PCR analysis of the murine and human flora.** Quantitative Real-Time-PCR amplifying bacterial 16S rRNA variable regions. 16S rRNA gene numbers/ ng DNA from luminal colon content from hfa (H) or mfa (M) mice after stable re-colonization before *C. jejuni*-infection (day 0) of the following bacterial groups were determined: *Enterobacteriaceae* (EB), Enterococci (EC), Lactic acid bacteria (LB). *Bacteroides/Prevotella* spp. (BP), *Clostridium leptum* group (CL), *Clostridium coccoides* group (CC), Mouse intestinal bacteroidetes (MIB), *Bifidobacteria* (BB), and total eubacterial load (TL). Numbers of animals harboring the respective bacterial rRNA are given in parentheses. Medians and significance levels (*P*-values) determined by Mann-Whitney-U test are indicated. Data shown were pooled from three independent experiments. **(C) Genetic fingerprinting of the murine and human flora.** Molecular fingerprints were generated by PCR-based DGGE analysis of total DNA isolated from luminal colon contents as described (see methods) of six individual hfa (left panel) and mfa mice (right panel). Samples were taken after stable recolonization, but before *C. jejuni*-infection (day 0) and 42 days after infection (day 42 p.i.).

Using our gnotobiotic mouse model we were able to study the dual interaction between *C. jejuni* and the murine host. In order to get deeper insights into the triangle relationship (“ménage à trois”) of the pathogen, the host innate immune system and the human versus the murine microbiota, we replenished gnotobiotic mice with a human or murine gut flora by gavage. Quantitative analyses of the main bacterial communities by culture (**Fig. 3 A**) and molecular techniques (**Fig. 3 B–C**, **Fig. S4**) revealed that the human or murine microbiota was effectively and permanently established within the gut for more than six weeks as independently confirmed by PCR-based genetic fingerprinting of fecal samples (**Fig. 3 C**). According to the typical differences in the microbiota composition of the two host species, lactobacilli were predominantly found in the murine flora associated (mfa) mice whereas human flora associated (hfa) animals harbored higher total bacterial and higher individual species loads of enterobacteria (*E. coli*), enterococci, *Bacteroides/ Prevotella spp.*, and clostridia as determined by cultural (**Fig. 3 A**) and molecular (**Fig. 3 B**) analyses of feces at d0 (right before *C. jejuni*-infection). A quantitative molecular six-weeks follow-up revealed that *C. jejuni*-infected “humanized” mice displayed higher enterobacteria, *Bacteroides/ Prevotella* spp. and *Clostridium coccoides* loads in their feces as compared to mfa animals (**Fig. S4 A**).

### *C. jejuni* colonization in “humanized” mice

**Figure 4:**
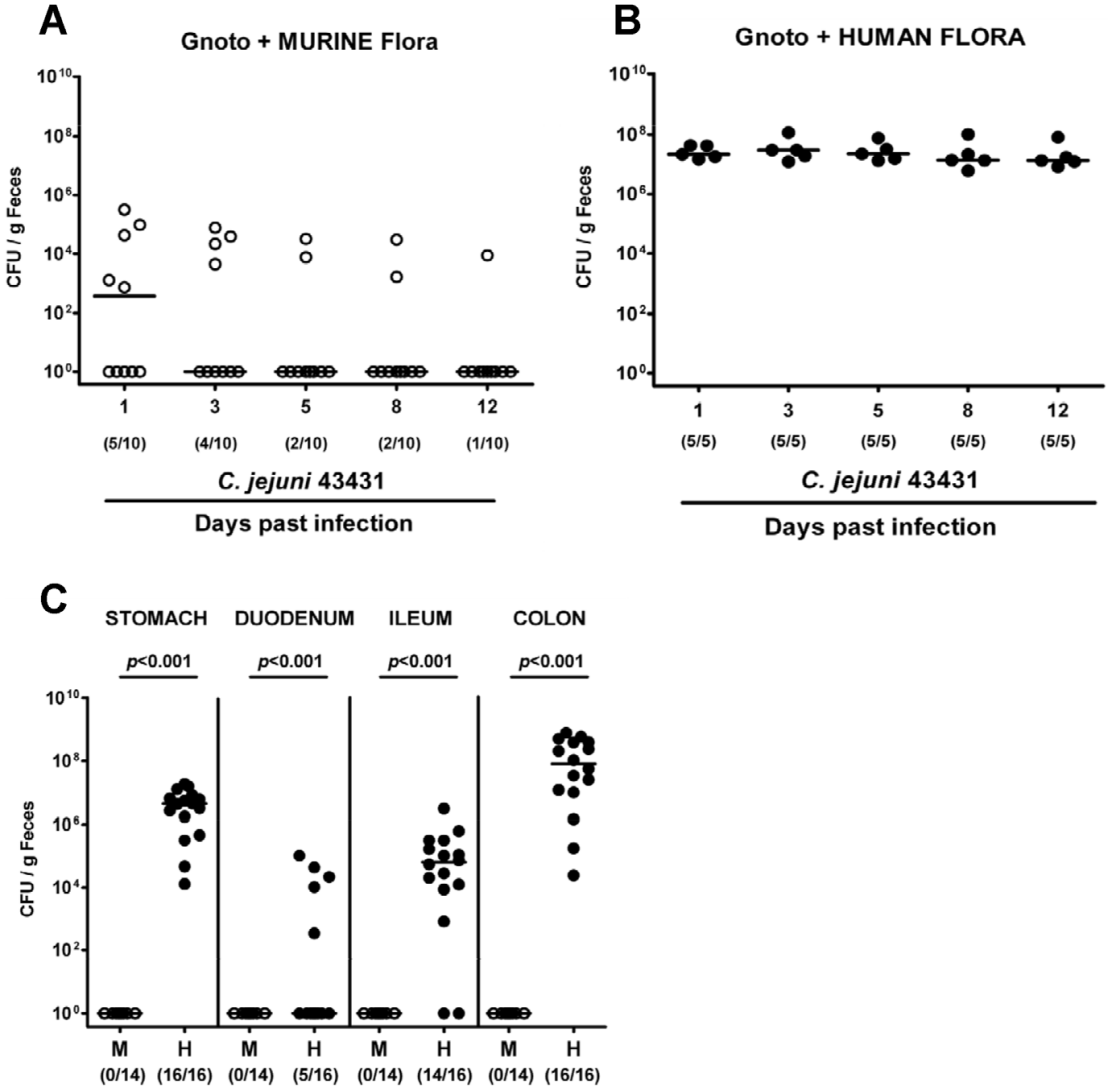
*C. jejuni* colonization in mice with a murine or human gut microbiota. Following oral infection with *C. jejuni* ATCC 43431, kinetic analyses of the pathogen loads in feces samples of gnotobiotic mice reconstituted with a murine (**A**) or human (**B**) gut flora until d12 p.i. were performed by culture (as described in methods; CFU, colony forming units). In addition, *C. jejuni* burdens in distinct compartments of the gastrointestinal tract (stomach, duodenum, ileum, colon; **C**) of mfa (M, open circles) and hfa (H, filled circles) mice at d12 p.i. were determined by quantification of live *C. jejuni* by culture. Numbers of animals harboring *C. jejuni* out of the total number of analyzed animals are given in parentheses. Medians (black bars) and significance levels (*P*-values, hfa as compared to mfa animals) determined by Mann-Whitney-U test are indicated. Data shown are representative for three independent experiments.

Two weeks following reconstitution of gnotobiotic mice with human or murine flora (to assure proper establishment of the microbiota along the GI tract), hfa and mfa mice were perorally infected with *C. jejuni* ATCC 43431 (d0). Kinetic analyses of the pathogen loads were performed in fecal samples and in distinct compartments of the entire GI tract after necropsy (d12 p.i.). Results revealed that mfa mice were protected from intestinal colonization as indicated by effective clearance of *C. jejuni* from the GI tract during the first two to three days p.i. (**Fig. 4 A**). In “humanized” mice, however, *C. jejuni* ATCC 43431 did stably colonize (**Fig. 4 B**) with bacterial loads of up to 10^8^ CFU per g feces. Long-term kinetic experiments in hfa mice revealed that *C. jejuni* ATCC 43431 colonization of stomach, ileum, and colon was stable for more than six weeks p.i. with the highest bacterial loads in the colon (**Fig. 4 C**).

### *C. jejuni*-induced intestinal immunopathology in “humanized” mice

**Figure 5:**
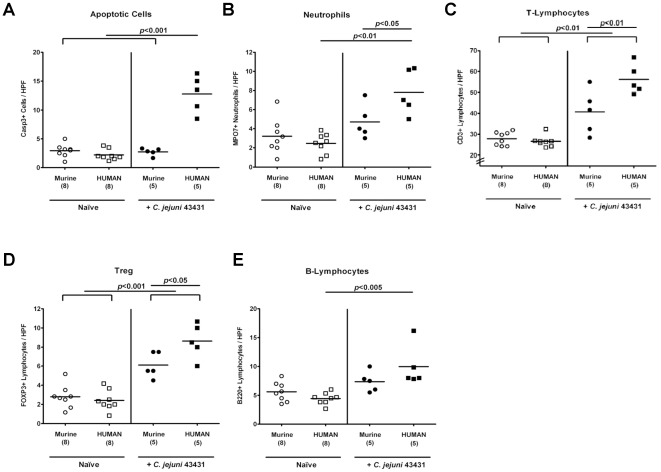
Immunopathological responses in the colon of “humanized” mice *in situ* following *C. jejuni*-infection. Mfa and hfa mice were generated and orally infected with *C. jejuni* strain ATCC 43431 by gavage as described in methods section. The average numbers of apoptotic cells (postive for caspase-3, panel **A**), neutrophilic granulocytes (neutrophils, positive for MPO-7, panel **B**), T-lymphocytes (positive for CD3, panel **C**), Tregs (positive for FOXP3, panel **D**), and B-lymphocytes (positive for B220, panel **E**) from at least six high power fields (HPF, 400× magnification) per animal were determined microscopically in immunohistochemically stained colon sections of naïve (Naïve, open symbols) and infected (+*C. jejuni* ATCC 43431, filled symbols) mfa (circles) and hfa (squares) mice at day 12 p.i.. Numbers of analyzed animals are given in parentheses. Medians (black bars) and levels of significance (*P*-values) as compared to the indicated groups (determined by Student's *t*-test) are indicated. Data shown are representative for three independent experiments.

In humans, *C. jejuni* induces the recruitment of pro-inflammatory immune cell populations to sites of inflammation in the colon [4], [5]. Therefore, we investigated apoptotic cell, neutrophil, T- and B-lymphocyte as well as Treg recruitment in the colonic mucosa during *C. jejuni*-infection in both, hfa and mfa mice by immunohistochemical staining of Caspase-3, MPO7, CD3, B220, and FOXP3 in colon sections of infected mice, respectively (**Fig. 5**). Remarkably, after reconstitution of gnotobiotic mice with a human or murine gut microbiota no differences were observed in the numbers of apoptotic cells, neutrophilic granulocytes, T- and B-lymphocytes or Tregs in the colon mucosa *in situ*. However, quantitative analyses of the respective immune cell populations after *C. jejuni* ATCC 43431-infection revealed that *C. jejuni*-infection induced a pronounced inflammatory response in the colon of hfa mice (**Fig. 5**). Twelve days p.i., a five-fold increase in apoptotic cells within the colon mucosa was observed in hfa mice as compared to infected mfa and uninfected control animals (**Fig. 5 A**). This increase of apoptotic cells was accompanied by a three-fold increase in neutrophil numbers in the colon mucosa of *C. jejuni*-infected hfa mice (**Fig. 5 B**). Furthermore, higher T- and B-lymphocyte as well as Treg counts were found in the colon mucosa at d12 following *C. jejuni*-infection with significantly higher T-lymphocyte and Treg numbers in hfa as compared to mfa mice (**Fig. 5 C–E**).

**Figure 6:**
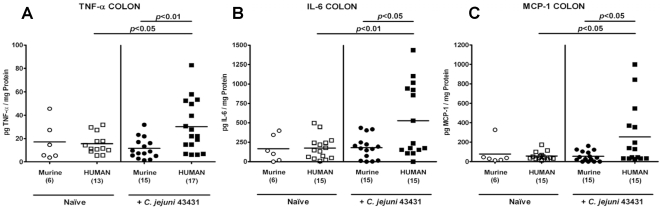
Pro-inflammatory cytokine responses in the colon of *C. jejuni*-infected “humanized” mice. Mfa and hfa mice were generated and orally infected with *C. jejuni* strain ATCC 43431 as described (see methods). Secretion of (**A**) TNF-α, (**B**) IL-6 and (**C**) MCP-1 was determined in supernatants of *ex vivo* colon cultures from naïve animals (Naïve, open symbols) or infected (+*C. jejuni* ATCC 43431, filled symbols) mfa (circles) or hfa (squares) mice at day 12 p.i.. Numbers of analyzed animals are given in parentheses. Means (black bars) and levels of significance (*P*-values) as compared to the indicated groups (determined by Student's *t*-test) are indicated. Data shown were pooled from two (for naïve mice) or three (for infected animals) independent experiments.

Next, we determined secretion of pro-inflammatory cytokines in the colon of *C. jejuni*-infected mice. At d12 p.i., hfa mice displayed higher TNF-α, IL-6 and MCP-1 concentrations in their large intestine as compared to mfa and uninfected control animals (**Fig. 6 A–C**). It is noteworthy that following *C. jejuni*-infection the respective means of TNF-α, IL-6, and MCP-1 levels increased by factors 2, 3, and 5, respectively, as compared to naïve hfa controls. The multi-fold increases of secreted pro-inflammatory cytokines, in the context of significant increases in immune cell numbers in colons sections of infected hfa mice (Fig. 5), point towards a biological relevant effect despite the high standard deviations within the infected hfa groups.

Taken together, following *C. jejuni*-infection the “humanized” mice are not only susceptible to colonization but also display typical pro-inflammatory features of human campylobacteriosis.

### The role of *C. jejuni* formic acid metabolism and perception in inducing immunopathology

**Figure 7:**
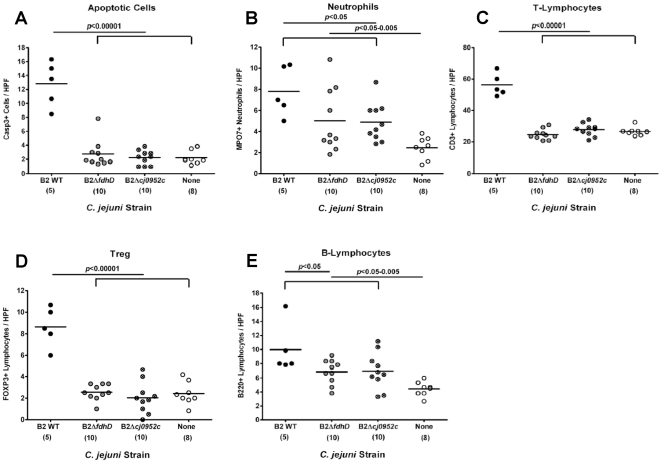
Impact of *C. jejuni* formic acid metabolism and perception on immunopathology in “humanized” mice. Hfa mice were generated as described (see methods) and orally infected with the *C. jejuni* wildtype strain (B2 WT), or with isogenic mutants deficient in the formate dehydrogenase subunit D (B2Δ*fdhD*) or the formic acid receptor (B2Δ*cj0952c*). The average numbers of apoptotic cells (positive for Caspase-3, panel **A**), neutrophilic granulocytes (neutrophils, positive for MPO-7, panel **B**), T-lymphocytes (positive for CD3, panel **C**), regulatory T-cells (Treg, positive for FOXP3, panel **D**) and B-lymphocytes (positive for B220, panel **E**) from at least six high power fields (HPF, 400× magnification) per animal were determined microscopically in immunohistochemically stained colon sections of naïve, non-infected (None, open circles) and infected (B2 WT: filled circles; B2Δ*fdhD*: grey circles; B2Δ*cj0952c*: crossed circles) mice at d12 post infection. Numbers of analyzed animals are given in parentheses. Means (black bars) and levels of significance (*P*-values) as compared to the respective groups (determined by Student's *t*-test) are indicated. Data shown are representative for three independent experiments.

We further investigated gnotobiotic and hfa mice as suitable experimental models for the study of *C. jejuni* virulence factors in colonization and immunopathology. For this purpose we performed infection experiments with two different *C. jejuni* mutant strains deficient in factors essential for cellular invasion. Both mutant strains were recently generated by transposon mutagenesis and displayed a reduced invasion capacity into epithelial cell lines *in vitro* [37]. The mutant strains carrying transposon (TnKan) insertions in the genes for formate dehydrogenase subunit D *fdhD* (strain B2Δ*fdhD*) or the formic acid receptor Tlp7 (strain B2Δ*cj0952c*) were generated in the *C. jejuni* B2 background as described earlier [37]. The formic acid receptor was shown to be associated with the cell invasion capacity of *C. jejuni* [37]. Briefly, if compared to the parental strain, the B2Δ*cj0952c* mutant displayed a five times reduced invasion capacity (as determined in gentamicin protection assays with Caco-2 cells). To investigate the role of formate dehydrogenase and the formic acid receptor in immunopathology we infected hfa mice with the B2Δ*fdhD* or B2Δ*cj0952c* mutant strain (**Fig. S5**). Control mice were infected with the parental *C. jejuni* strain B2. Results indicate that the invasion capacity and the formic acid metabolism are not required for intestinal colonization, but play essential roles in *C. jejuni*-mediated immunopathology. Both mutant strains colonized hfa mice similarly at high loads (up to 10^7^–10^8^ CFU/ g feces) as compared to the parental B2 strain (**Fig. S5 A–C**). Immunopathology, however, was exclusively induced by the parental strain, but not by either mutant (**Fig. 7**). Numbers of apoptotic cells, T-lymphocytes and Tregs within the colon mucosa 12 days after infection with either mutant remained comparable to non-infected mice, but highly significantly increased multi-fold after oral infection with the parental strain (**Fig. 7 A, C, D**). In addition, the numbers of neutrophils and B-lymphocytes increased in the colon of hfa mice infected with either mutant strain compared to non-infected controls (**Fig. 7 B, E**). However, the MPO7^+^ or B220^+^ cell counts were higher in animals infected with the parental strain as compared to mice infected with either mutant.

**Figure 8:**
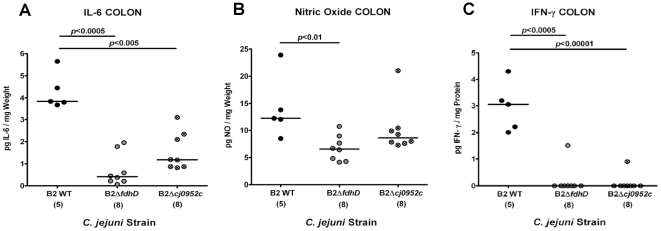
Impact of *C. jejuni* formic acid metabolism and perception on cytokine responses in “humanized” mice. Hfa mice were generated as described (see methods) and orally infected with the *C. jejuni* wildtype strain (B2 WT), or with isogenic mutants deficient in the formate dehydrogenase subunit D (B2Δ*fdhD*) or the formic acid receptor (B2Δ*cj0952c*). Secretion of (**A**) IL-6, (**B**) NO and (**C**) IFN-γ was determined in supernatants of *ex vivo* colon cultures obtained from infected (B2 WT: filled circles; B2Δ*fdhD*: grey circles; B2Δ*cj0952c*: crossed circles) mice at d12 p.i.. Numbers of analyzed animals are given in parentheses. Means (black bars) and levels of significance (*P*-values) as compared to the respective groups (determined by Student's *t*-test) are indicated. Data shown are representative for three independent experiments.

Furthermore, lower numbers of *in situ* immune cell counts were accompanied by significantly lower IL-6 and IFN-γ protein concentrations in *ex vivo* colon cultures obtained from mice infected with the respective mutant as compared to the parental strain infected controls (at d12 p.i.). In addition, mice infected with the *C. jejuni* mutant B2Δ*fdhD* displayed lower nitric oxide secretion in the colon as compared to mice infected with the parental strain (**Fig. 8 A–C**).

### *C. jejuni* translocation in gnotobiotic and hfa, but not mfa mice

**Figure 9:**
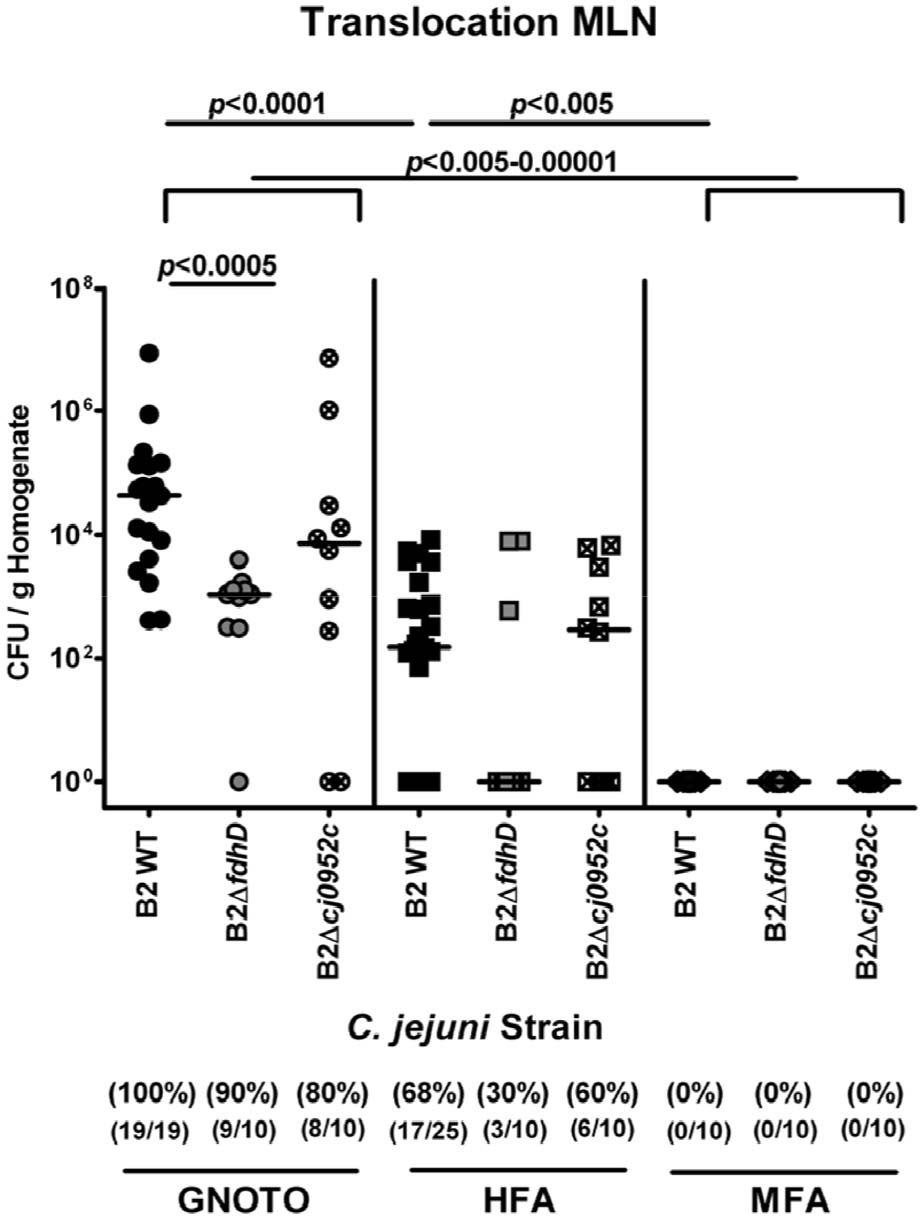
Impact of *C. jejuni* formic acid metabolism and perception on bacterial translocation to mesenteric lymphnodes. Gnotobiotic (GNOTO; circles) and with human flora associated (HFA; squares) mice were generated as described (see methods). Conventionally colonized (MFA; diamonds) animals served as negative controls. Mice were orally infected with the *C. jejuni* wildtype strain (B2 WT; solid symbols), or with isogenic mutants deficient in the formate dehydrogenase subunit D (B2Δ*fdhD*; grey symbols) or the formic acid receptor (B2Δ*cj0952c*; crossed symbols). At d12 post infection, live *C. jejuni* were quantified in homogenized MLNs by culture (as described in methods; CFU, colony forming units). Medians (black bars) and significance levels (*P*-values) determined by Mann-Whitney-U test are indicated. Absolute and relative numbers of animals harboring *C. jejuni* are given in parentheses. Data shown are pooled from three independent experiments.

Studies have shown that live *C. jejuni* is able to translocate to the mesenteric lymph nodes (MLNs) in infected isolator-raised germfree animals [12]–[14]. Therefore, we determined the translocation capacity of the parental strain and both mutants, B2Δ*fdhD* and B2Δ*cj0952c in vivo*. Quantitative cultural analysis of live bacteria in MLNs, spleen, liver, kidney, and cardiac blood of infected gnotobiotic and hfa mice revealed that at d12 p.i. *C. jejuni* translocates to MLNs (**Fig. 9**) but not to other organs or blood (data not shown) in gnotobiotic or hfa mice. Mfa mice displaying a strong colonization resistance against *C. jejuni* served as negative controls. Interestingly, the translocation frequencies (100% vs. 68%) as well as the bacterial loads (medians of approximately 10^5^ vs. 10^2^ CFU/ g organ homogenate) of the *C. jejuni* B2 parental strain in MLNs were higher in infected gnotobiotic versus “humanized” animals at day 12 p.i., with no differences as compared to other *C. jejuni* strains such as ATCC 43431 and 81–176 (not shown). In gnotobiotic and hfa mice, both, the *C. jejuni* B2Δ*fdhD* and B2Δ*cj0952c* mutant strain, translocated less frequently into MLNs as compared to the parental strain with lower counts of approximately two orders of magnitude following infection with the B2Δ*fdhD* mutant *versus* the parental strain (**Fig. 9**).

## Discussion

The commensal intestinal flora constitutes a barrier to effectively prevent the host from colonization and infection by gut pathogens. The earlier observation that *C. jejuni* readily colonized isolator-raised germfree mice [13]–[15] pointed towards an essential role of the conventional gut microbiota in the colonization resistance against *C. jejuni*. The results presented here do further underline the impact of the murine microbiota in colonization resistance as was independently confirmed by effective *C. jejuni*-infection of gnotobiotic, but not mfa mice. In comparison with isolator-raised germfree mice, we favor our animal model for the following reasons: First, gnotobiotic mice generated by antibiotic treatment exhibit a physiologically developed immune system. Second, these mice can be generated under standardized breeding conditions in a sterile environment within a common animal facility and without the need for special equipment (such as isolators for instance) and, thus, costs for generating and housing are significantly lower.

An experimental model of a human pathogen in mice previously depleted of their intestinal gut microbiota and subsequently replenished with human gut flora allows the study of host-pathogen interactions in the natural host environment. Given that these “humanized” mice, but not mice with a murine flora, are susceptible to *C. jejuni*-infection indicates for the first time, that the host-specific microflora composition is essential for susceptibility or resistance to *Campylobacter*-infections resembling the natural host conditions. However, individual bacterial species or the complex microflora composition responsible for colonization resistance have not been identified so far and await further detailed investigation.

The susceptibility of gnotobiotic and hfa mice to *C. jejuni*-infection renders the novel models presented here excellently suited for standardized and reproducible analyses of *C. jejuni* colonization and immunopathology in a higher vertebrate experimental system. Most importantly, the inflammatory responses in the colon of *C. jejuni*-infected “humanized” mice *in situ* mimicked key features of immunopathological responses in human campylobacteriosis [4], [5]: First, tissue damage was most pronounced in the lower intestinal tract as indicated by increased numbers of apoptotic cells in the colon mucosa. Second, *C. jejuni*-induced mucosal injury was accompanied by an increased recruitment of innate immune and effector cells (e.g. T- and B-lymphocytes, Tregs and neutrophils) in the colon mucosa of infected mice. Lastly, an increased secretion of pro-inflammatory cytokines such as TNF-α, IL-6, and MCP-1 were found in *ex vivo* colon cultures taken from infected hfa mice. Interestingly, Treg numbers increased in the colons of hfa mice during the course of *C. jejuni*-infection suggesting that production of IL-10 and other anti-inflammatory mediators might be involved in limiting *C. jejuni*-induced immunopathology. This was further supported by Bell and coworkers (2009) who showed that *C. jejuni* is able to induce a strong inflammatory response in IL-10 deficient mice [27].

Using our gnotobiotic infection model we could further dissect the interplay between *C. jejuni* and the host innate responses. Infection of gnotobiotic mice deficient in MyD88, TRIF or TLR4 revealed for the first time *in vivo* that MyD88- and TRIF-dependent TLR4-recognition of bacterial LPS is essentially involved in the development of immunopathology during *C. jejuni* enteritis as indicated by lower numbers of apoptotic cells, neutrophils, T- and B-lymphocytes as well as Tregs found in the colon mucosa of the respective gene-deficient mice at day 12 p.i.. Similarly, other *in vitro* studies have shown the important role of TLR4-signaling in *C. jejuni* LPS infected cells [22] and activation of human TLR4 by *C. jejuni* [21].

Strikingly, our results obtained from infected TLR9-deficient mice for the first time point towards a pivotal role of bacterial CpG-DNA in mediating *C. jejuni*-induced immunopathology. The activation of innate immune cells via TLR9, however, depends on the cell type, and on both, the methylation status and sequence of the DNA ligand under study [39]. Furthermore, a selected set of *Campylobacter* strains did not activate human TLR9 expressed in a transfected human cancer cell line [21]. However, this does not rule out a role for TLR9 in human campylobacteriosis. The *C. jejuni* population is genetically highly variable and could present DNA of diverging sequence or methylation status to host immune cells in a strain dependent manner. The *C. jejuni* strains used for analysis of campylobacteriosis in mice presented here were neither studied for TLR9 activation in human nor murine cell lines before. Thus, the fact that *C. jejuni*-infected gnotobiotic mice lacking TLR9 displayed less severe immunopathology points towards an important role of TLR9 in the murine model. The role of TLR9 in human *C. jejuni*-infection awaits further confirmation by additional studies with cells of the adaptive and innate immune system.

Also noteworthy is the contribution of bacterial DNA and TLR9 signaling in augmenting murine intestinal inflammation supported by our previous studies: In murine intestinal graft-versus-host disease we observed recently that TLR9 is essentially involved in aggravation of colitis [40].

Finally, the murine *C. jejuni*-infection models presented here are valuable tools to investigate *C. jejuni* virulence factors *in vivo*. Hfa mice infected with *C. jejuni* mutant strains deficient in formic acid metabolism or perception involved in cell invasion fitness harbored comparable bacterial loads throughout the GI tract. However, both mutant strains significantly displayed less immunopathological responses as indicated by lower numbers of apoptotic cells, neutrophils, T-lymphocytes, and Tregs in the colon mucosa as compared to mice infected with the parental strain. This diminished cellular response was paralleled by lower expression of pro-inflammatory cytokines (IL-6, nitric oxide, and IFN-γ) and less translocation of living *C. jejuni* into MLNs in animals infected with the B2Δ*fdhD* or B2Δ*cj0952c* strain as compared to mice infected with the wildtype strain. Given that these results underline the suitability of the animal model presented here for *in vivo* analyses of *C. jejuni* gene mutant strains, there is a possibility of polar effects on downstream genes which cannot be ruled out. Thus, the relevance of the *fdhD* or the *cj0952c* gene mutation for amelioration of immunopathology and translocation following oral infection cannot unequivocally be attributed to the gene products encoded by the deleted genes. Nevertheless, since formic acid is a metabolite produced by many commensal gut bacteria and thus part of the intestinal intraluminal milieu, these findings point towards an essential role of formic acid metabolism and perception in the intestinal “lifestyle” of *C. jejuni* and its cell invasiveness *in vivo*.

Taken together, we conclude that our novel murine models for colonization and inflammation following *C. jejuni*-infection shown in the present study, mimic many important aspects of human *C. jejuni*-infection and thus offer promising novel tools to better understand the arousing “ménage à trois” between the pathogen, commensal gut bacteria, and host innate immune responses.

## Materials and Methods

### Ethics Statement

All animal experiments were conducted according to the European Guidelines for animal welfare (2010/63/EU) with approval of the commission for animal experiments headed by the “Landesamt für Gesundheit und Soziales” (LaGeSo, Berlin, Germany; registration number TVV G0173/07). Animal welfare was monitored twice daily by assessment of clinical conditions. Fresh human fecal samples for recolonizing gnotobiotic mice (hfa) were collected from healthy volunteers. Before sample collection written informed consent was obtained from all volunteers. Since fecal samples were obtained from co-workers of our laboratory and thus outside a clinical environment and used for re-colonization of mice only, experiments were exempted from approval by the Charité - Universitätsmedizin ethical committee according to German legacy (§15, Legal Basis for Clinical Trials).

### Mice

All animals were maintained in the facilities of the “Forschungsinstitut für Experimentelle Medizin” (FEM, Charité - Universitätsmedizin, Berlin, Germany), under specific pathogen-free (SPF) conditions. Mice deficient in TLR4 (C57BL/10ScSn (C10) background), TLR9, TRIF or MyD88 (C57BL/6 (C6) background), used in the experiments are described in detail elsewhere [40]. Age and sex matched mice between 10 and 12 weeks of age were used.

### Generation of gnotobiotic mice

To eradicate the commensal gut flora, mice were transferred to sterile cages and treated by adding ampicillin (1 g/L; Ratiopharm), vancomycin (500 mg/L; Cell Pharm), ciprofloxacin (200 mg/L; Bayer Vital), imipenem (250 mg/L; MSD), and metronidazole (1 g/L; Fresenius) to the drinking water ad libitum for 6–8 weeks as described earlier [30].

### Generation of gnotobiotic mice with a human or murine gut flora

Fresh human and murine fecal samples free of enteropathogenic bacteria, parasites, and viruses were collected from five individual healthy volunteers and animals, respectively, pooled and dissolved in an equal volume of sterile PBS, aliquoted and stored at −80°C until use. For reconstitution experiments, aliquots were thawed and bacterial communities quantified by cultural and molecular methods (refer to [30]) before gavage of mice with 0.3 mL of the respective suspension. Between independent experiments bacterial counts of groups varied of less than 0.5 log orders of magnitude.

### *C. jejuni*-infection of mice

Conventional, gnotobiotic or mice reconstituted with human or murine gut flora were infected with 10^9^ viable CFU of *C. jejuni* strains ATCC 43431, 81–176, B2 or mutant strains B2Δ*fdhD* and B2Δ*cj0952c* deficient in the formate dehydrogenase subunit D or the formic acid receptor gene, respectively [37], by gavage in a total volume of 0.3 mL PBS on three consecutive days.

### Sampling procedures and histopathology

Mice were sacrificed by isofluran treatment (Abbott, Germany). Cardiac blood and tissue samples from liver, spleen, kidneys, MLNs, and gastrointestinal (GI) tract (stomach, duodenum, ileum, colon) were removed under sterile conditions. GI samples from each mouse were collected in parallel for histological, microbiological, immunological, and molecular analyses. Histopathological changes were determined in colon samples immediately fixed in 5% formalin and embedded in paraffin. Sections (5 µm) were stained with hematoxylin and eosion (HE) and examined by light microscopy (magnification ×200).

### Immunohistochemistry

*In situ* immunohistochemical analysis of colon paraffin sections was performed as described previously [40]. Primary antibodies against CD3 (#N1580, Dako, Denmark, dilution 1∶10), myeloperoxidase-7 (MPO-7, # A0398, Dako, 1∶10000), FOXP-3 (FJK-16s, eBioscience, 1∶100), B220 (eBioscience, San Diego, CA, USA, 1∶200), and cleaved caspase-3 (Asp175, Cell Signaling, USA, 1∶200) were used. For each animal, the average number of positively stained cells within at least six high power fields (HPF, 400× magnification) were determined microscopically by three independent investigators (MMH, RP, AAK).

### Quantitative analysis of *C. jejuni* translocation

Live *C. jejuni* were detected in MLNs, spleen, liver, kidneys, and cardiac blood by culture. Tissues were homogenized in sterile PBS and analyzed in dilution series on karmali agar (Oxoid, Wesel, Germany) in a microaerophilic atmosphere at 37°C for at least 48 hours. Cardiac blood (0.2 mL) was immediately streaked out on karmali agar plates.

### Analysis of the intestinal microflora

Cultural analyses, biochemical identification, and molecular detection of luminal bacterial communities from stomach, duodenum, ileum, and colon were performed as previously described [30], [31], [41]. Genetic fingerprints of the intestinal microflora were generated by PCR-based denaturing-gradient-gel-electrophoresis (PCR-DGGE) amplifying the 16S rRNA variable region V6–8 [30]. Quantitative real-time PCR (qRT-PCR) was performed on a StepOnePlus RealTime-PCR System (Applied Biosystems) using the Maxima SYBR Green/ROX qPCR Master Mix (Fermentas). The reaction volume was 20 µl using 2 µl template DNA at following cycling conditions (40 cycles): Initial denaturation / enzyme activation at 95°C for 10 min, initial denaturation at 95°C for 15 s followed by annealing / elongation at 60°C for 1 min. Fluorescence detection was performed at 80°C (75°C for BP/MIB; 70°C for EC) after each cycle. A melting curve for each run was performed to check for the correct amplicon. Total bacteria (TL), gamma-*Proteobacteria* / *Enterobacteriaceae* (EB), *Lactobacillus* group (LB), *Clostridium leptum* group (CL), *Clostridium coccoides* group (CC), *Bacteroides* group including *Prevotella* and *Porphyromonas* (BP), Mouse Intestinal *Bacteroidetes* (MIB), Enterococci (EC) and *Bifidobacteria* (BB) were separately detected and quantified using 16S primer sets described earlier [40]. The *C. jejuni* loads of feces samples and luminal GI contents were determined with a flagellin A based qRT-PCR (*flaA* AW 50 fw, 5′-ATGGGATTTCGTATTAACAC-3′, *flaA* AW 5 re 5′-GATATAGCTTGACCTAAAGTA-3′) resulting in a 197bp product (primers taken from: Dr. K. Pietsch, Freiburg, personal communication). The *Campylobacter*-*flaA*-PCR cycling conditions were different to the above mentioned: Annealing was at 55°C for 20 s, elongation at 60°C for 30 s and fluorescence detection at 70°C. In general, the 16S rRNA or *flaA* gene numbers / ng DNA of each sample were determined, not the actual bacterial numbers.

### Cytokine detection in colon culture supernatants

Colon biopsies were cut longitudinally, washed in PBS, and strips of 1 cm^2^ were placed in 24-flat-bottom well culture plates (Nunc, Wiesbaden, Germany) containing 500 µl serum-free RPMI 1640 medium supplemented with penicillin (100 U/ ml) and streptomycin (100 ug/ ml; PAA Laboratories). After 18 h at 37°C, culture supernatants were tested for TNF-α, IL-6, MCP-1, and IFN-γ by the Mouse Inflammation Cytometric Bead Assay (CBA; BD Biosciences) on a BD FACSCanto II flow cytometer (BD Biosciences). Nitric oxide (NO) was determined by Griess-reaction as described earlier [30].

### Statistical analysis

Mean values, medians, standard deviations, and levels of significance were determined using appropriate tests as indicated (two-tailed Student's *t*-Test, Mann-Whitney-U Test). Two-sided probability (*P*) values≤0.05 were considered significant. All experiments were repeated at least twice.

## Supporting Information

Figure S1 **PCR-based detection of gut bacteria in feces from conventionally colonized, but not gnotobiotic mice.** PCR analysis with eubacterial primer amplifying 16S V6–8 region of DNA from fecal samples obtained from nine conventionally raised mice (conventional) and gnotobiotic mice (gnotobiotic) were performed and run on 1% agarose gels stained with ethidiumbromide.
(TIF)

Figure S2 ***C. jejuni*** **colonization in gnotobiotic and conventional mice.** Conventional wildtype mice (SPF, open circles), and gnotobiotic mice (GNOTO, filled circles, generated by antibiotic gut decontamination) were orally infected with *C. jejuni* strains 81–176, ATCC 43431 or B2 (as indicated on the x-axis) as described (see methods). The colonization capacity was determined by quantification of live *C. jejuni* in feces samples at day 12 p.i. by cultural analysis (CFU, colony forming units). Numbers of animals harboring *C. jejuni* out of the total number of analyzed animals are given in parentheses. Medians (black bars) and significance levels (*P*-values, as compared to SPF animals) determined by Mann-Whitney-U test are indicated. Data shown were pooled from three independent experiments.
(TIF)

Figure S3 **Histopathology in colon sections following** ***C. jejuni*** **infection.** Paraffin sections of colon samples were HE-stained as described (see methods). (A) In naïve, uninfected wildtype mice many goblet cells (dashed arrows), normal crypt architecture, and no immune cell infiltration were observed. (B) TLR4-deficient mice displayed mild immune cell infiltration (solid arrow), whereas in wildtype animals (C) loss of goblet cells (dashed arrows), crypt elongation, and moderate immune cell infiltration into the lamina propria (solid arrow) could be detected at day 12 following *C. jejuni* ATCC 43431-infection. Representative photomicrographs (magnification ×200) from three independent experiments are shown.
(TIF)

Figure S4 **Quantitative molecular analysis of fecel samples obtained from mfa and hfa mice.** (**A**) Quantitative Real-Time-PCR amplifying bacterial 16S rRNA variable regions. 16S rRNA gene numbers / ng DNA from luminal colon content of mfa (M, open squares) or hfa (H, filled squares) recolonized mice after *C. jejuni*-infection (day 42 p.i.) of the following bacterial groups were determined: *Enterobacteriaceae* (EB), enterococci (EC), lactic acid Bacteria (LB), *Bacteroides/ Prevotella* spp. (BP), *Clostridium leptum* group (CL), *Clostridium coccoides* group (CC), mouse intestinal bacteroidetes (MIB), *Bifidobacteria* (BB), and total eubacterial load (TL). Medians (black bars) and significance levels (*P*-values) determined by Mann-Whitney-U test are indicated. Quantitative loads of the respective bacterial groups at day 0 (after stable re-colonization; circles) and at day 42 (d42, squares) after *C. jejuni*-infection obtained from MFA (**B**; open symbols) and HFA (**C**; filled symbols) mice were analyzed by qRT-PCR. Numbers of animals harboring the respective bacterial rRNA are given in parentheses. Medians are indicated as black bars. Data shown were pooled from three independent experiments.
(TIF)

Figure S5 **Impact of** ***C. jejuni*** **formic acid metabolism and perception on colonization in “humanized” mice.** Human flora associated mice (hfa) were generated as described (see methods) and orally infected with the *C. jejuni* wildtype (WT; **A**) strain B2, or with isogenic mutants deficient in the formate dehydrogenase subunit D (B2Δ*fdhD*; **B**) or the formic acid receptor (B2Δ*cj0952c*; **C**). The kinetic analysis of colonization capacity was determined by quantification of live *C. jejuni* in luminal colon samples until day 12 post infection by culture (CFU, colony forming units). Medians (black bars) and days post infection (on x-axis) are indicated. Numbers of animals harboring *C. jejuni* out of the total number of analyzed animals are given in parentheses. Data shown were pooled from three independent experiments.
(TIF)
