# Correction: Novel Murine Infection Models Provide Deep Insights into the “Ménage à Trois” of *Campylobacter jejuni*, Microbiota and Host Innate Immunity

**DOI:** 10.1371/annotation/5247af81-4595-44b7-9c3f-2e45ad85abfa

**Published:** 2011-08-23

**Authors:** Stefan Bereswill, André Fischer, Rita Plickert, Lea-Maxie Haag, Bettina Otto, Anja A. Kühl, Javid I. Dashti, Andreas E. Zautner, Melba Muñoz, Christoph Loddenkemper, Uwe Groß, Ulf B. Göbel, Markus M. Heimesaat

The 7th author's name is misspelled. The correct 7th author's name is: Javid I. Dasti 

