# Supplementary figures and images for: Novel Murine Infection Models Provide Deep Insights into the “Ménage à Trois” of Campylobacter jejuni, Microbiota and Host Innate Immunity
Source: PLoS One. 2011 Jun 15;6(6):e20953. doi: 10.1371/journal.pone.0020953 (PMC3115961; doi:10.1371/journal.pone.0020953)

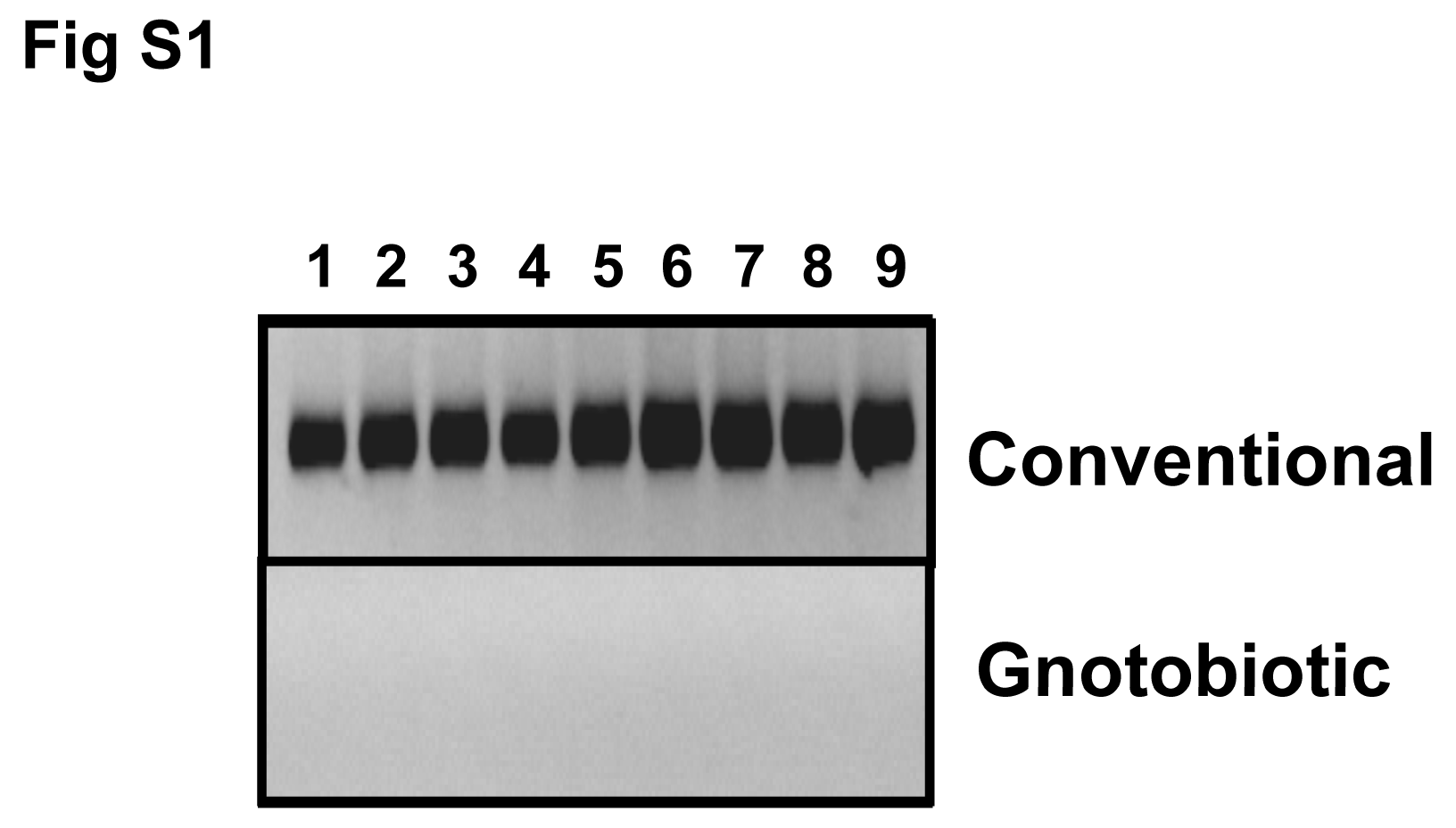

Supplement: Figure S1 — PCR-based detection of gut bacteria in feces from conventionally colonized, but not gnotobiotic mice. PCR analysis with eubacterial primer amplifying 16S V6–8 region of DNA from fecal samples obtained from nine conventionally raised mice (conventional) and gnotobiotic mice (gnotobiotic) were performed and run on 1% agarose gels stained with ethidiumbromide. (TIF) [file pone.0020953.s001.tif]

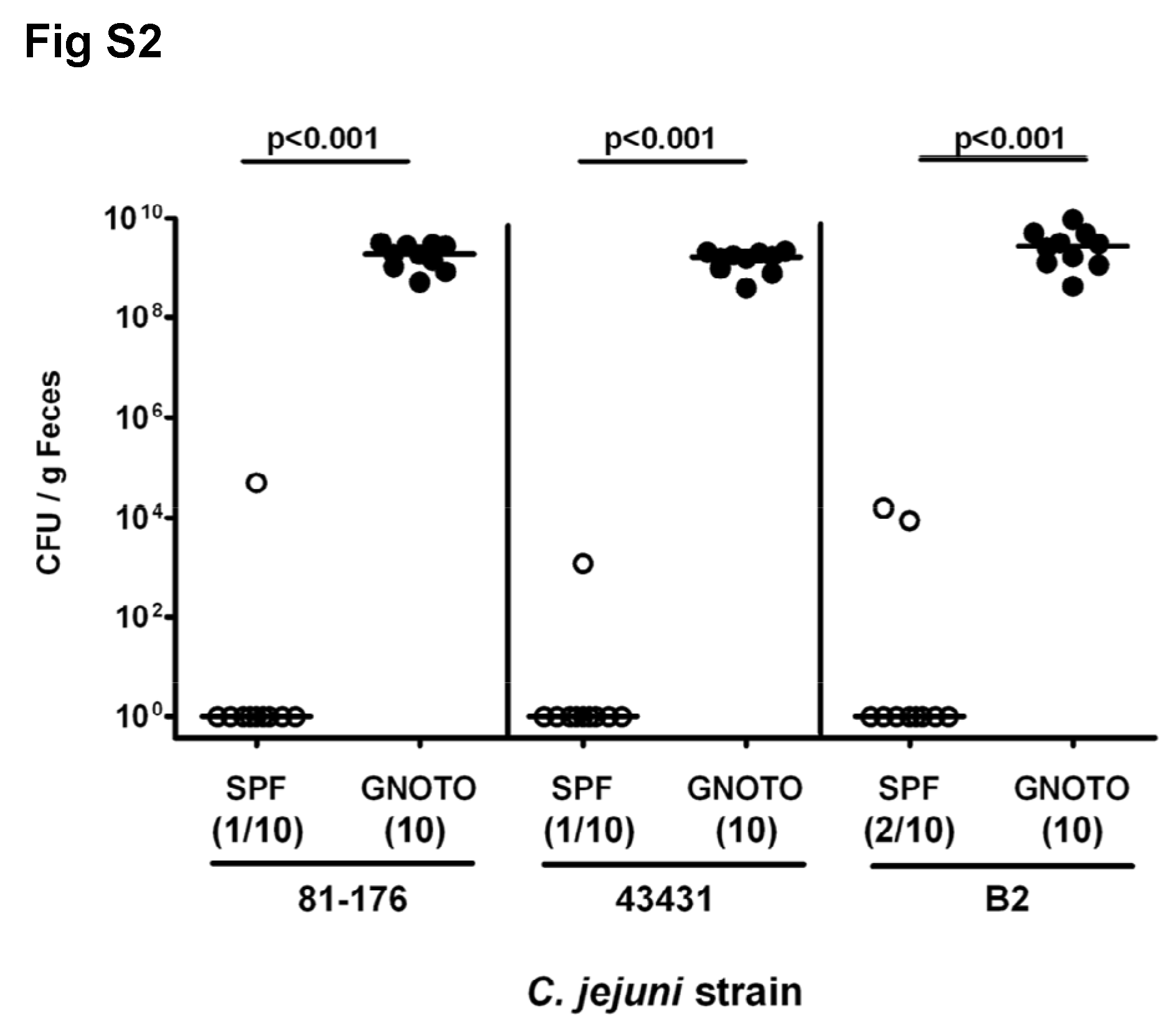

Supplement: Figure S2 — C. jejuni colonization in gnotobiotic and conventional mice. Conventional wildtype mice (SPF, open circles), and gnotobiotic mice (GNOTO, filled circles, generated by antibiotic gut decontamination) were orally infected with C. jejuni strains 81–176, ATCC 43431 or B2 (as indicated on the x-axis) as described (see methods). The colonization capacity was determined by quantification of live C. jejuni in feces samples at day 12 p.i. by cultural analysis (CFU, colony forming units). Numbers of animals harboring C. jejuni out of the total number of analyzed animals are given in parentheses. Medians (black bars) and significance levels (P-values, as compared to SPF animals) determined by Mann-Whitney-U test are indicated. Data shown were pooled from three independent experiments. (TIF) [file pone.0020953.s002.tif]

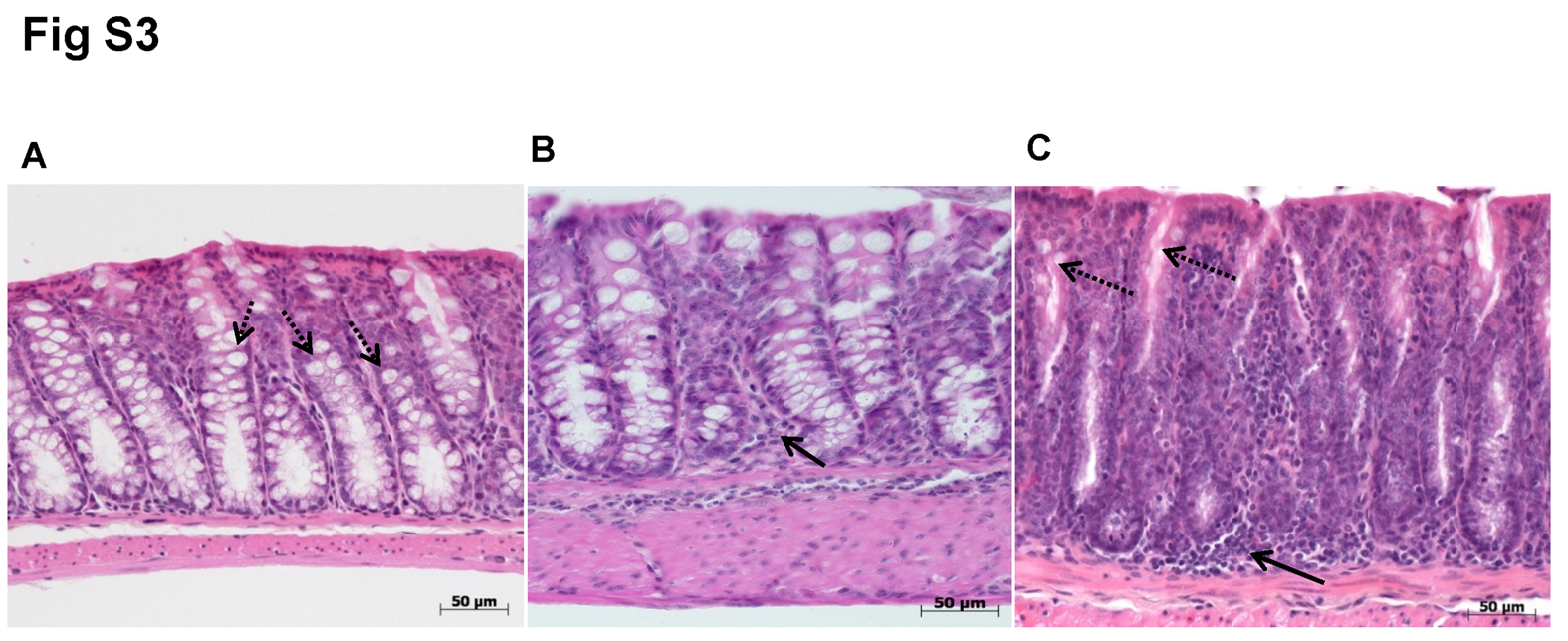

Supplement: Figure S3 — Histopathology in colon sections following C. jejuni infection. Paraffin sections of colon samples were HE-stained as described (see methods). (A) In naïve, uninfected wildtype mice many goblet cells (dashed arrows), normal crypt architecture, and no immune cell infiltration were observed. (B) TLR4-deficient mice displayed mild immune cell infiltration (solid arrow), whereas in wildtype animals (C) loss of goblet cells (dashed arrows), crypt elongation, and moderate immune cell infiltration into the lamina propria (solid arrow) could be detected at day 12 following C. jejuni ATCC 43431-infection. Representative photomicrographs (magnification ×200) from three independent experiments are shown. (TIF) [file pone.0020953.s003.tif]

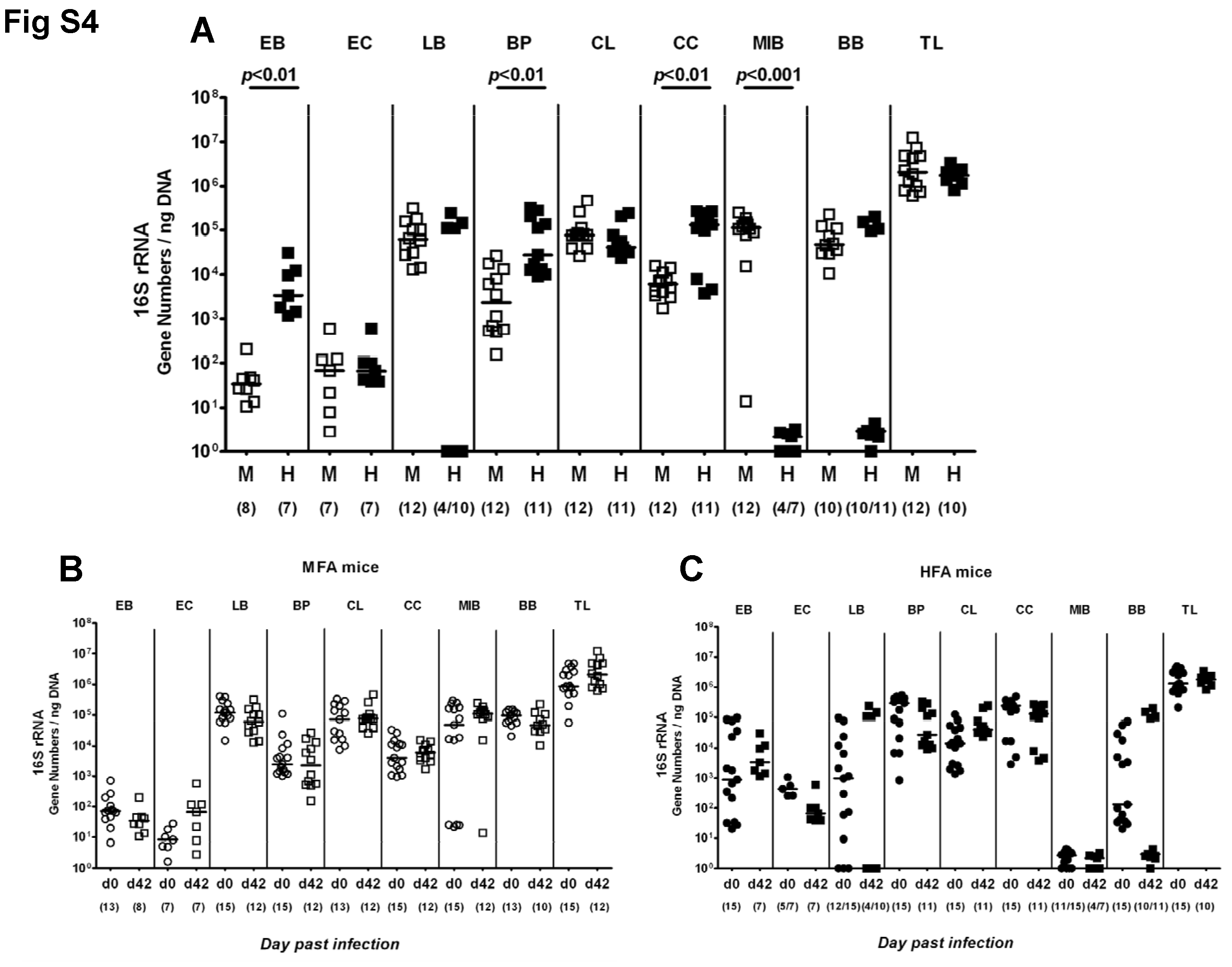

Supplement: Figure S4 — Quantitative molecular analysis of fecel samples obtained from mfa and hfa mice. (A) Quantitative Real-Time-PCR amplifying bacterial 16S rRNA variable regions. 16S rRNA gene numbers / ng DNA from luminal colon content of mfa (M, open squares) or hfa (H, filled squares) recolonized mice after C. jejuni-infection (day 42 p.i.) of the following bacterial groups were determined: Enterobacteriaceae (EB), enterococci (EC), lactic acid Bacteria (LB), Bacteroides/ Prevotella spp. (BP), Clostridium leptum group (CL), Clostridium coccoides group (CC), mouse intestinal bacteroidetes (MIB), Bifidobacteria (BB), and total eubacterial load (TL). Medians (black bars) and significance levels (P-values) determined by Mann-Whitney-U test are indicated. Quantitative loads of the respective bacterial groups at day 0 (after stable re-colonization; circles) and at day 42 (d42, squares) after C. jejuni-infection obtained from MFA (B; open symbols) and HFA (C; filled symbols) mice were analyzed by qRT-PCR. Numbers of animals harboring the respective bacterial rRNA are given in parentheses. Medians are indicated as black bars. Data shown were pooled from three independent experiments. (TIF) [file pone.0020953.s004.tif]

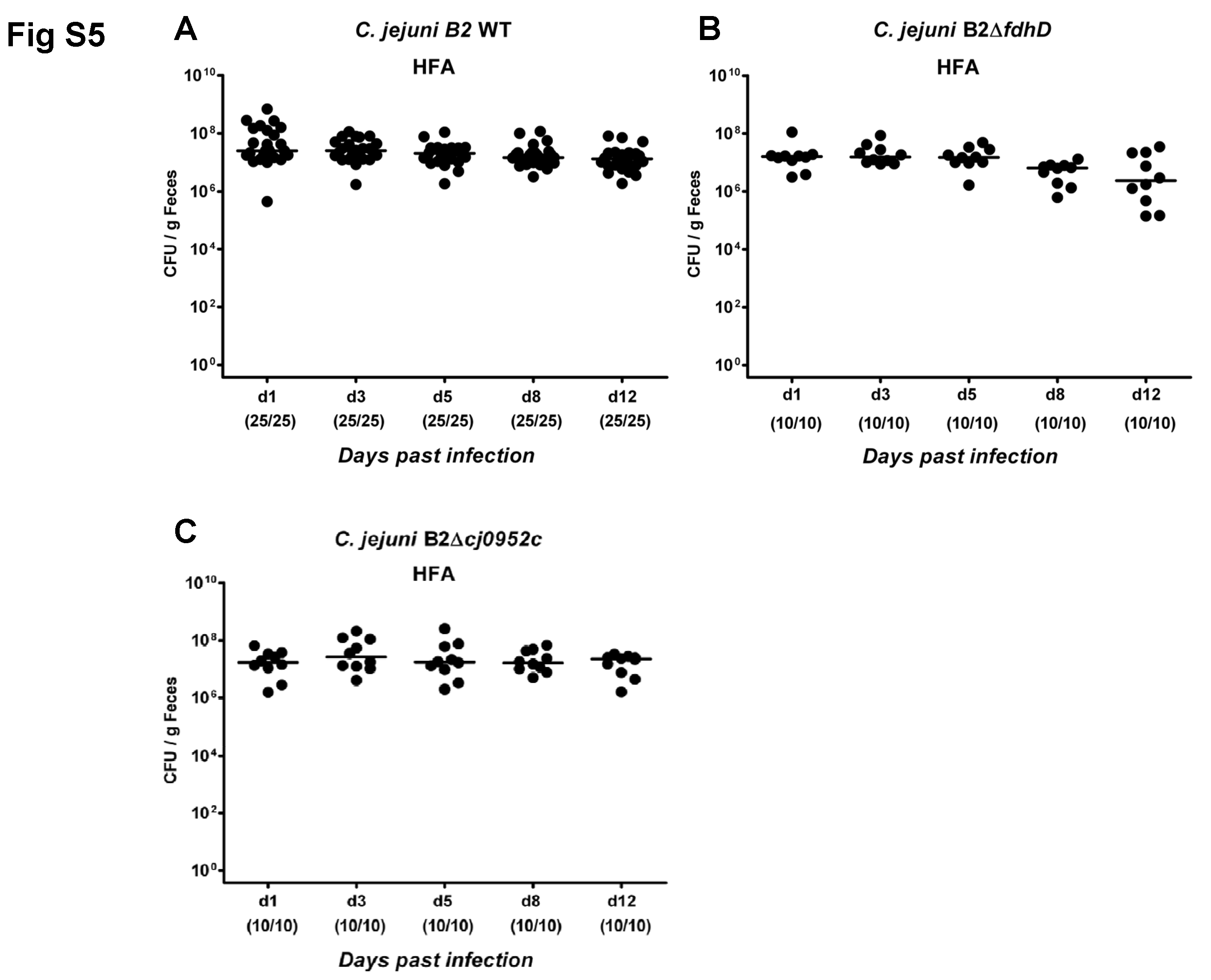

Supplement: Figure S5 — Impact of C. jejuni formic acid metabolism and perception on colonization in “humanized” mice. Human flora associated mice (hfa) were generated as described (see methods) and orally infected with the C. jejuni wildtype (WT; A) strain B2, or with isogenic mutants deficient in the formate dehydrogenase subunit D (B2ΔfdhD; B) or the formic acid receptor (B2Δcj0952c; C). The kinetic analysis of colonization capacity was determined by quantification of live C. jejuni in luminal colon samples until day 12 post infection by culture (CFU, colony forming units). Medians (black bars) and days post infection (on x-axis) are indicated. Numbers of animals harboring C. jejuni out of the total number of analyzed animals are given in parentheses. Data shown were pooled from three independent experiments. (TIF) [file pone.0020953.s005.tif]
